# The Micropaleoecology Framework: Evaluating Biotic Responses to Global Change Through Paleoproxy, Microfossil, and Ecological Data Integration

**DOI:** 10.1002/ece3.70470

**Published:** 2024-10-31

**Authors:** Adam Woodhouse, Anshuman Swain, Jansen A. Smith, Elizabeth C. Sibert, Adriane R. Lam, Jennifer A. Dunne, Alexandra Auderset

**Affiliations:** ^1^ School of Earth Sciences University of Bristol Bristol UK; ^2^ University of Texas Institute for Geophysics University of Texas at Austin Austin Texas USA; ^3^ Department of Organismic and Evolutionary Biology Harvard University Cambridge Massachusetts USA; ^4^ Museum of Comparative Zoology Harvard University Cambridge Massachusetts USA; ^5^ Department of Earth and Environmental Sciences University of Minnesota Duluth Duluth Minnesota USA; ^6^ Department of Geology and Geophysics Woods Hole Oceanographic Institution Woods Hole Massachusetts USA; ^7^ Department of Earth Sciences Binghamton University Binghamton New York USA; ^8^ Santa Fe Institute Santa Fe New Mexico USA; ^9^ School of Ocean and Earth Science University of Southampton Southampton UK

**Keywords:** biogeochemistry, biomarkers, climate change, conservation paleobiology, ecosystems, IODP, microfossils, oceanography, paleoceanography, paleontology

## Abstract

The microfossil record contains abundant, diverse, and well‐preserved fossils spanning multiple trophic levels from primary producers to apex predators. In addition, microfossils often constitute and are preserved in high abundances alongside continuous high‐resolution geochemical proxy records. These characteristics mean that microfossils can provide valuable context for understanding the modern climate and biodiversity crises by allowing for the interrogation of spatiotemporal scales well beyond what is available in neo‐ecological research. Here, we formalize a research framework of “micropaleoecology,” which builds on a holistic understanding of global change from the environment to ecosystem level. Location: Global. Time period: Neoproterozoic‐Phanerozoic. Taxa studied: Fossilizing organisms/molecules. Our framework seeks to integrate geochemical proxy records with microfossil records and metrics, and draws on mechanistic models and systems‐level statistical analyses to integrate disparate records. Using multiple proxies and mechanistic mathematical frameworks extends analysis beyond traditional correlation‐based studies of paleoecological associations and builds a greater understanding of past ecosystem dynamics. The goal of micropaleoecology is to investigate how environmental changes impact the component and emergent properties of ecosystems through the integration of multi‐trophic level body fossil records (primarily using microfossils, and incorporating additional macrofossil data where possible) with contemporaneous environmental (biogeochemical, geochemical, and sedimentological) records. Micropaleoecology, with its focus on integrating ecological metrics within the context of paleontological records, facilitates a deeper understanding of the response of ecosystems across time and space to better prepare for a future Earth under threat from anthropogenic climate change.

## Graphical abstract

The microfossil record is the most complete archive of ancient ecosystem response to past climate change (e.g., Aze et al. 2011). It contains abundant, diverse, and well‐preserved fossils spanning multiple trophic levels from primary producers to apex predators which often constitute and are preserved in high abundances alongside continuous high‐resolution geochemical proxy records. These characteristics can be paired with analytical metrics to provide valuable fossil context for understanding the modern climate and biodiversity crises, a research framework which we formalize here as “micropaleoecology”.

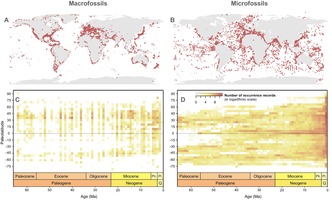

## 1 Introduction

One of the great societal challenges of the 21st century is adapting to and mitigating the consequences of anthropogenic climate change. Extreme, rapid change driven by anthropogenic activities impacts both the environment (e.g., CO_2_‐induced global warming, changes in nutrient distributions) and ecosystems (e.g., biodiversity loss due to over‐fishing and hunting, land‐use changes). The impacts of specific climate forcing on individual species, as well as feedbacks between environmental and biological changes at different scales, can have major add‐on effects on the structure and function of natural systems that can push ecosystems past tipping points, leading to abrupt shifts to contrasting ecosystem states (Dakos et al. 2019) and, in some cases, wholesale ecological collapse (Brook, Sodhi, and Bradshaw 2008). These cascading changes not only impact the constituent organisms and their interactions but also the broader‐scale ecosystem functions and services that are vital resources for human existence (IPBES 2019).

Teasing out the impacts of environmental change on ecosystem dynamics is a complex problem that requires interdisciplinary solutions and the best available data. Ecosystems function at multiple spatial and temporal scales with interacting biotic and abiotic components. Although the records of these dynamics are variable in availability and quality, they are essential for constructing a comprehensive understanding of how past, present, and future versions of the Earth system function under different climate regimes.

Here, we develop a framework for investigating climate‐ecosystem dynamics throughout the geological record that explicitly leverages microfossil data. This framework, which we call “micropaleoecology,” unites the exceptional quality and coverage of multi‐trophic level micropaleontological records with geochemical paleo‐proxy climatic reconstructions using neo‐ecological metrics (e.g., Hill numbers (Hill 1973), co‐occurrence (Morales‐Castilla et al. 2015)) and systems‐level methods (e.g., network‐based methods (Bascompte 2007) and agent‐based models (Grimm 1999)). The potential value of the micropaleoecology framework lies in the ability to investigate ecosystem‐wide changes, from primary producers to consumers across all trophic levels, in relation to past environmental shifts. The high temporal and spatial resolution and coverage of the microfossil and biogeochemical records, combined with abiotic environmental datasets, provides a holistic approach to infer linkages between ecosystems and external forcing mechanisms. This interdisciplinary research framework can contribute to the understanding of ecosystem sensitivity and the potential impacts of environmental and biological change (Hunt, Cronin, and Roy 2005; Yasuhara et al. 2012, 2017a, 2017b, 2020a, 2020b; Moffitt et al. 2015; Chiu et al. 2017; Cramer et al. 2017; Myhre et al. 2017; Schmidt 2018; Doi, Yasuhara, and Ushio 2021).

## 2 Records of Biotic and Abiotic Change

Many datasets focus on restricted data types (e.g., biotic *or* abiotic; neontological *or* paleontological; taxon‐specific rather than community‐based), perpetuating challenges for developing a long‐term, ecosystem‐wide perspective. For example, the extent of long‐term ecological studies is largely confined to the last 50 years (Dornelas et al. 2018), with an average study window of 3–5 years (Estes et al. 2018) and sampling intervals of days, weeks, or months. However, despite the accelerated rate and magnitude of the current climate crisis in comparison with geological crises, many “modes” of variability in modern ecosystems are decadal or longer in scale and not captured with these short time series (e.g., Dietl et al. 2015; Mantua and Hare 2002; Eguchi et al. 1999, 2003; Gupta et al. 2002; Hull, Darroch, and Erwin 2015; Tsutsui et al. 2016). The patterns detected in these modern studies provide a snapshot of ecosystems that is valuable in its own right but many “unsolved problems in ecology” (see Dobson, Tilman, and Holt 2020) require broader spatial and deeper temporal perspectives that can be gained by viewing these short‐term trajectories within the scope of the longer processes of which they are a part (Dietl et al. 2015; Lazarus 1994). Moreover, the majority of modern observations began within a climate system already altered by anthropogenic forcing (McCulloch et al. 2024), highlighting the need for biological datasets that effectively capture background states and variability before system perturbations.

**FIGURE 1:**
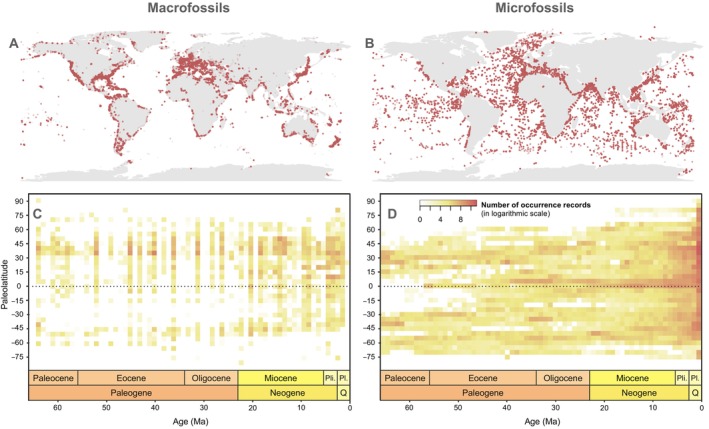
Geographical occurrence records of (A) marine macrofossils (from Paleobiology Database) and (B) microfossils (combined records from BioDeepTime, Triton and Sibert et al. 2014, 2016, 2020 and unpublished data). (C) and (D) show the same records on a paleolatitudinal‐time axis of 1‐million‐year time bins and 5° paleolatitudinal bands.

One of the best resources for interrogating the long‐term relationship between life and the environment is the microfossil record. Microfossils—defined here as fossils requiring the use of a microscope for study—provide broad geographic coverage through space and time (Armstrong and Brasier 2013). Microfossils encompass nearly all trophic levels, from primary producers (e.g., diatoms, calcareous nannofossils/coccolithophores, pollen) to top predators (e.g., fish teeth, shark dermal denticles, mammal teeth), and many groups in between (e.g., foraminifera, ostracodes, fungi). The high preservation potential of microfossils allows them to be collected, sometimes in great numbers, from outcrop sections and sediment cores from ocean and lake environments, in many cases at high temporal resolution due to their nearly uninterrupted deposition in aquatic basins (Figure 1; see BioDeepTime, Smith et al. 2023a; Smith et al. 2023b; Marsaglia et al. 2015; IODP Science Support Office, 2018). Such excellent spatiotemporal coverage has led to microfossil‐based studies addressing questions about evolutionary rates, biodiversity hotspots, and biogeographic patterns with temporal durations ranging from seasonal to multi‐million year, on temporal scales from the Precambrian to Recent, and on geographic scales from local to global (e.g., de Vernal and Hillaire‐Marcel 2008; Alvarez et al. 2019; Trubovitz et al. 2020; Pilarczyk et al. 2014; Lam and Leckie 2020; Lowery et al. 2020; Yasuhara et al. 2017a, 2017b, 2020a, 2020b, 2022; Nowak, Schneebeli‐Hermann, and Kustatscher 2019; Jamson, Moon, and Fraass 2022; Song et al. 2011; Lowery and Fraass 2019; Schopf 1993; Riedman et al. 2014; Fenton et al. 2016a, 2016b, 2023; Jonkers et al. 2019; Sibert et al. 2018; Sibert and Rubin 2021; Mottl et al. 2021; Woodhouse et al. 2023b; Woodhouse et al. 2021, 2023a; Swain, Woodhouse et al. 2024; Aze et al. 2011).

Large paleontological datasets (e.g., GBDB (Fan et al. 2013), Neotoma (Williams et al. 2018), Neptune (Renaudie et al. 2020), DINOSTRAT (Bijl 2022), Paleobiology Database (Uhen et al. 2023), and Triton (Fenton, Woodhouse et al. 2021)) are invaluable resources for evaluating past global biodiversity change and can be used to assess ecological and evolutionary features of past climate and environmental perturbations, such as size selectivity during extinctions (Rego et al. 2012), biogeographic range shifts (Fenton et al. 2023; Woodhouse et al. 2023a), and changes in trophic dynamics (Smith et al. 2022; Woodhouse et al. 2023b; Swain 2023). However, not all data in these large accumulations lend themselves to high‐resolution, multi‐trophic‐level ecological reconstruction. For example, the macrofossil record occasionally offers high species richness, large abundances, and exceptional preservation, supporting important insights into ancient ecosystems (e.g., Dunne et al. 2014), but such records are very much the exception, often occurring in geographically isolated lagerstätte (Benton 1989; Kidwell and Flessa 1995; Benton et al. 2011; Cisneros et al. 2022). Indeed, compared to microfossils, macrofossils tend to have small population sizes, low preservation potential, and are primarily preserved in transient depositional environments with irregular occurrences in Earth's history (e.g., Benton 1989; Benton et al. 2011).

**FIGURE 2:**
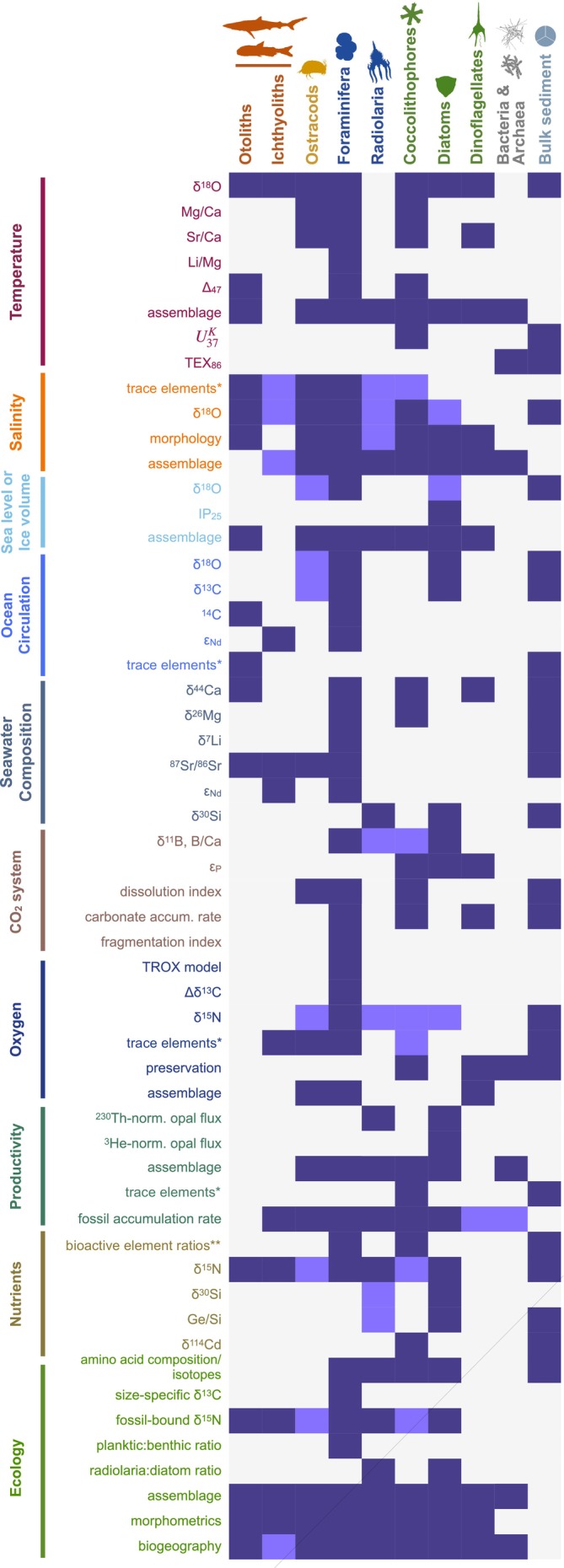
A dominantly marine‐based selection of (bio)geochemical & ecological proxies and their respective archives to reconstruct various environmental parameters. Dark purple fields indicate existing studies (see Supporting Information for specific references), light purple fields indicate potential measurements based on the geochemical proxy which can be applied to different archives in the future, grey fields indicate no application so far. * = e.g., Na/Ca, Mg/Ca, Sr/Ca etc., ** = e.g., Ca/Cd, Ca/Ba, Ca/Zn.

Complementary to the microfossil record, organic “molecular” fossils, including biomarkers and ancient DNA, can provide more information on some fossil organisms (e.g., ${U^{K}}_{37^{\prime }}$ from coccolithophores) as well as direct evidence of organisms with soft anatomies that are unlikely to fossilize (e.g., picoeukaryotes). These non‐fossilizing organisms play a fundamental role in marine ecosystems through photosynthesis and recycling of organic matter. Molecular fossils allow for high‐resolution biologic, paleoceanographic, and paleoclimatic analyses, and are broadly applicable to various aquatic and terrestrial environments (e.g., Sepúlveda et al. 2009; Lupien et al. 2022; Cluett et al. 2023), and ancient DNA analyses can provide comprehensive data on ancient terrestrial and marine community dynamics on multi‐million year timescales in exceptional circumstances (Kuwae et al. 2020; Armbrecht et al. 2022; Kjær et al. 2022; Nakamura et al. 2023). Molecular fossils can be used to reconstruct past sea surface temperatures (e.g., GDGTs/ TEX_86_, Schouten et al. 2002), oxygen conditions in the water column and sediment (e.g., pristane/phytane ratio or bulk sedimentary nitrogen isotopes, Rontani et al. 2003; Altabet and Francois 1994), or heterotrophic vs. autotrophic carbon cycling type (Figure 2; also see list of biomarkers in Volkman et al. 1998). These fossils are especially useful in reconstructing spatial and temporal nutrient availability, including essential nutrients like nitrogen and phosphorus (Redfield 1958), which ultimately drive ecosystem productivity and the overarching structure of microfossil communities (e.g., alkenones and chlorins, Harris et al. 1996; Raja and Rosell‐Melé 2021). Furthermore, there are dozens of non‐skeletal, sediment‐bound inorganic proxies that preserve records of environmental conditions (e.g., X‐ray fluorescence (Croudace and Rothwell 2015), and natural gamma radiation (Adams and Gasparini 1970)).

A wide array of geochemical proxies derived from microfossils, molecular fossils, and bulk sediments (Table S1; Figure 2) have additionally been developed to study climate‐ecosystem interactions through further elucidation of paleoclimatic/paleoceanographic (e.g., temperature and CO_2_), paleoenvironmental (e.g., nutrient and oxygen concentrations), and paleoecological (e.g., biogenic barium) conditions. Such records have unveiled the changing nature of Earth's ice volume and temperature through the Cenozoic (e.g., Zachos et al. 2001, 2008; Cramer et al. 2009, 2011; Westerhold et al. 2020), Mesozoic (e.g., Trotter et al. 2015; Huber et al. 2018), and Paleozoic (e.g., Trotter et al. 2008; Quinton et al. 2018). Records of oceanic conditions have been reconstructed from geochemical proxies, such as pH estimated from B/Ca ratios in planktic foraminifera (e.g., Sanyal et al. 1995; Yu et al. 2007; Foster and Rae 2016), past ocean circulation as estimated from neodymium isotopes preserved in fish teeth (e.g., Martin and Haley 2000; Dera et al. 2009; Huck et al. 2016; Thomas et al. 2014; Kender et al. 2018), and primary productivity from nitrogen isotopes obtained from a menagerie of microfossils or bulk sediment (e.g., Sigman et al. 1999) (Figure 2). Such records of abiotic system changes are informative in their own right, and have provided insight into the patterns of Antarctic ice growth and decay (e.g., Patterson et al. 2014), surface ocean circulation shifts (e.g., Lam et al. 2021); and atmospheric changes (e.g., Groeneveld et al. 2017), highlighting how such systems respond to times of increased atmospheric CO_2_ concentrations and global warmth. In addition, results from climate and ocean models can add another dimension to the analyses when combined with fossil studies (e.g., Lam, Stigall, and Matzke 2018; Lam, Sheffield, and Matzke 2021). The microfossil and molecular fossil record becomes even more informative when integrated with such environmental and geochemical proxy records through innovative usage of system‐level analytical methods (e.g., network analyses, dynamical system models, etc.). Such syntheses can elucidate biotic and abiotic changes over durations that encapsulate the complex and long‐acting processes at work within ecosystems (e.g., de Vernal and Hillaire‐Marcel 2008; Westerhold et al. 2020; Mottl et al. 2021; Woodhouse et al. 2022; Hou et al. 2023; Sepúlveda et al. 2009).

Traditionally, work on geochemical, paleoclimatic, and paleoenvironmental reconstructions has operated independently from ecological and evolutionary questions, despite the same physical samples being used and complementary data being produced. Integration of paleontological and geochemical datasets is critical to developing a more comprehensive understanding of the ecological responses to environmental change. Global efforts to investigate the rich geochemical environmental proxy and microfossil datasets are facilitated by the 55+ year legacy of scientific ocean drilling, which provides an excellent foundation of exceptional spatiotemporal scale on which to build an ecosystem‐wide view of life's interactions with environmental changes—in short, the perfect place to explore the micropaleoecology framework.

## 3 Micropaleoecology, a Synthesis of Perspectives

The goal of the micropaleoecology framework is to investigate how environmental changes impact the component and emergent properties of ecosystems through the integration of multi‐trophic level body fossil records (primarily using microfossils, though incorporating macrofossils where possible) (Figure 1) with contemporaneous molecular, geochemical, and sedimentological records across local and global scales (Figure 2). Concomitant analyses of data from each of these sources in a framework motivated by modern ecological and Earth system concepts will enable a more comprehensive view of Earth's ecosystem dynamics, particularly when also harnessing advances in big data collation and high‐performance computing.

By defining the framework of micropaleoecology, we seek to form a nucleus of theory and methodology that brings together ideas and researchers working in similar but often siloed disciplines. Similar interdisciplinary syntheses have grown in the past decade. For example, conservation paleobiology has brought together paleontologists and conservation biologists working on conservation issues (Dietl et al. 2015; Dillon et al. 2022), and archaeoecology provides an intellectual home for ecologists, archaeologists, and paleontologists seeking to understand the impact of humans on ecosystems since our species evolved (Crabtree and Dunne 2023). Similarly, micropaleoecology brings together micropaleontologists, (bio)geochemists, oceanographers, ecologists, and paleontologists (to name a few) working to disentangle the complex interactions between organisms and the environment. Although not defined formally, research that we would consider micropaleoecology has been ongoing, even if not previously named as such (e.g., Hunt, Cronin, and Roy 2005; Yasuhara et al. 2012; Moffitt et al. 2015; Chiu et al. 2017; Cramer et al. 2017; Myhre et al. 2017; Doi, Yasuhara, and Ushio 2021), and the term “micropaleoecology” has occasionally appeared in the literature, where it was passingly—and convergently—used to describe the intersectionality of microorganisms and environment (e.g., Wignall 1990; Elicki 2006; Diz et al. 2018; Hebda et al. 2022). An interdisciplinary approach that cross‐cuts traditional research boundaries will unlock and unite existing literature and datasets to more comprehensively investigate the interplay among the multiple climatic, environmental, and biological variables shaping the Earth and its ecosystems through time.

To date, there is a dearth of studies that combine biotic with abiotic records to fully capture and investigate the intricacies of the Earth system. Prior efforts by biologists and paleontologists have created biological‐focused databases to synthesize disparate neontological and paleontological datasets (e.g., Lazarus 1994; Fenton, Woodhouse et al. 2021; Williams et al. 2018; Bijl 2022; Smith et al. 2023a; Smith et al. 2023b; Sessa et al. 2023). Similarly, working groups composed of (paleo)oceanographers, paleoclimatologists, geochemists, Earth systems modelers, and sedimentologists have worked to synthesize records of abiotic changes, often with a focus on improving climate models (e.g., Haywood et al. 2011; Judd et al. 2022). Although many individual biotic and abiotic records are available from data aggregators (e.g., PANGAEA, Diepenbroek et al. 2002), there is a lack of standardization or ability to aggregate and collate these disparate datasets easily, but some efforts have been made (e.g., Sessa et al. 2023).

To compile and aggregate such disparate databases, harmonization across metrics and fields is essential; however, this process is complicated by many idiosyncrasies of the data sources, including challenges relating to nomenclature, age models, database structure, and communication. For use in taxon‐based ecological analyses, maintaining and monitoring synonymization of taxonomy across timescales and regions is a critical step for the collation of data from different time periods, geographic regions, and disciplines (e.g., Mottl et al. 2021). Reporting how age models are developed, along with raw data, precise sample depths and localities, and associated datum age and depth uncertainties, are essential for linking the different types of data (e.g., biological, environmental, geochemical). Standardization and homogenization of biotic and abiotic database structures require cross‐talk between experts to establish common terminology, methods, and quality standards (e.g., sample resolution, age model quality), synonymize database field names, and build database structures that can accommodate the nuances inherent to each data type. We share an example of such an interdisciplinary synonymized database structure that is able to accommodate both paleoceanographic and paleontological data in an integrated way (see Supporting Information). Lastly, to realize the full potential of such a comprehensive database and gain insights across timescales throughout Earth history, increased discussions and collaborations must take place between researchers interested in geo‐ and ecological sciences including, but not limited to geochronology, paleobiology, paleoclimate, paleoceanography, and ecology.

## 4 Building the Micropaleoecology Framework

The concept of integrating fossil records with environmental change dates back centuries (e.g., Parkinson 1822). The micropaleoecology framework builds on this concept from the organism to ecosystem level, by integrating geochemical proxy records with (micro)fossil records and metrics, drawing on mechanistic and systems‐level statistical analyses to link between the records. Although it is impossible to reconstruct every aspect of an ancient ecosystem, the integration of organism groups across multiple trophic levels (including less commonly used fossils like fish otoliths, ichtyoliths, ostracods, and bacteria) with multiple paleoproxies simultaneously allows for multi‐variate deconstruction of drivers of environmental, ecological, and evolutionary change: a true ecological view of past ecosystems.

**FIGURE 3:**
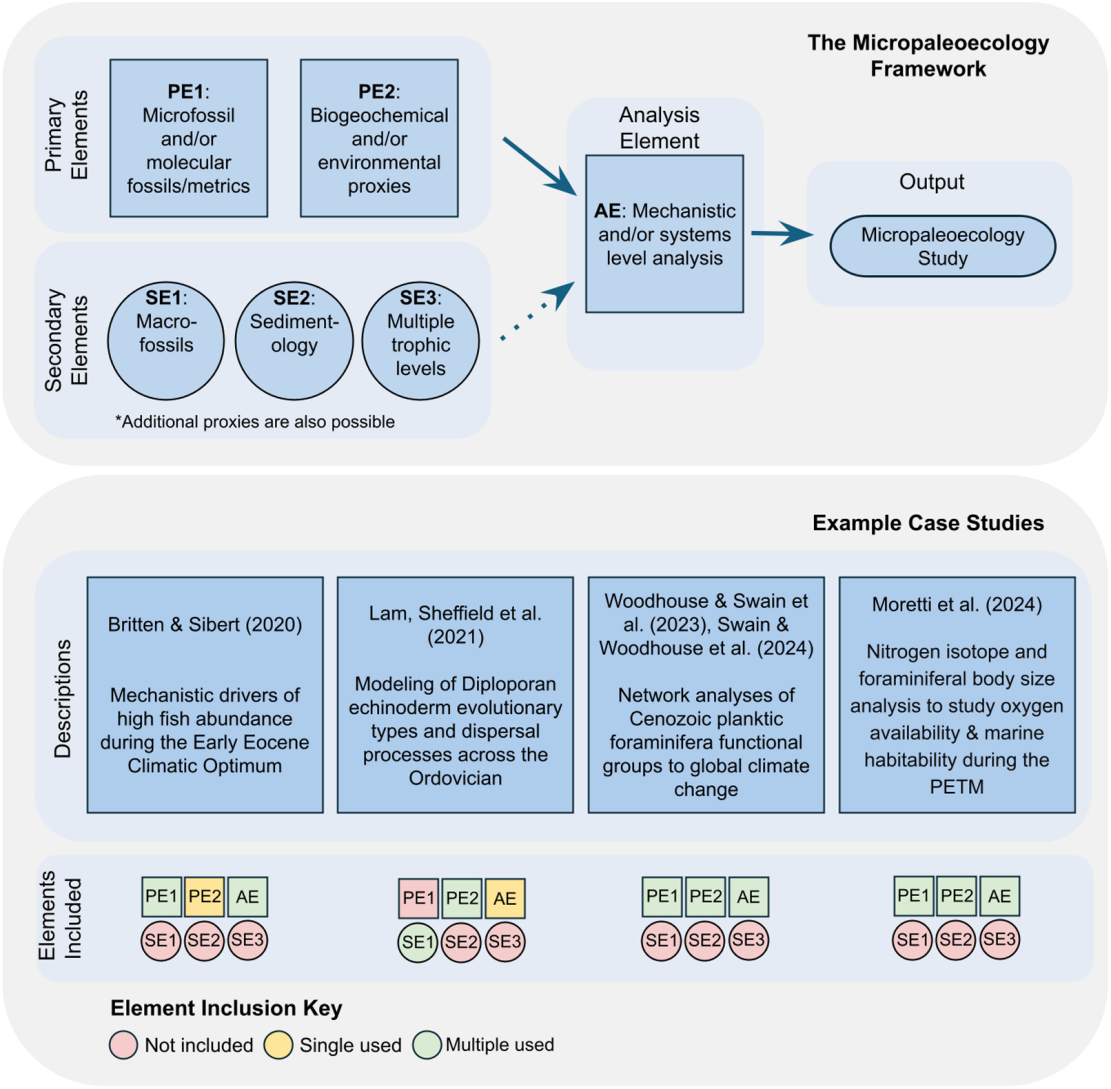
An overview of the proposed micropaleoecology framework with example case studies. An ideal micropaleoecology study includes data from multiple taxonomic groups and biogeochemical data, evaluated in an analytical framework informed by ecological theory with mechanistic models or systems‐level statistical analyses. AE, analysis element; PE, primary element; SE, secondary element.

Even though the micropaleoecology framework has not yet been applied in full form, its basic ideas and methods have been used in previous studies (see Figure 3). Notable examples include the work of Britten and Sibert (2020), who provided an organism group‐specific example of a multi‐metric, systems‐based approach investigating mechanistic drivers of high fish abundance during the Early Eocene Climate Optimum by integrating paleo‐temperature records and within‐assemblage fish tooth size distributions. To do so, they applied a size‐structured trophic transfer model and determined that elevated fish abundance during this interval was driven by increases in trophic transfer efficiency in pelagic marine ecosystems with extreme warmth (Britten and Sibert 2020).

Lam, Sheffield, and Matzke (2021) implemented phylogenetically‐informed paleobiogeographic analyses to infer dispersal paths and speciation types across the Ordovician for the diploporan echinoderms. Using biogeographic stochastic mapping, they inferred that founder‐event speciation was critical to the evolution of the group through time, and that dispersal between Baltica and the mid‐continent of Laurentia became more prevalent from the early to late Ordovician. A lack of correlation among speciation events, sea level, atmospheric oxygen levels, temperature, and carbon cycle changes indicate diploporans were responding to additional abiotic processes. Although this study focused on macrofossils, it exemplifies the type of research we advocate for in the micropaleoecology framework and the potential beneficial inclusion of additional information from the fossil record (e.g., macrofossils), when possible.

Woodhouse et al. (2023b) demonstrated the application of a network‐based approach that can be used to good effect in the micropaleoecology framework. They used this approach to analyze a dataset of planktic foraminifera with a high spatiotemporal resolution and identified co‐occurrence and specialization patterns of foraminiferal species in the last ~15 million years through a lens of trait‐based ecology and morphology. The results demonstrated that in planktic foraminifera, even though the latitudinal biodiversity gradient has remained invariant for the past 15 million years (i.e., tropics and near‐tropics are species‐rich), the areas where most ecological niches coexisted equitably have changed drastically across climatic conditions (Woodhouse et al. 2023b).

Moretti et al. (2024) integrated geochemical proxy records (nitrogen isotopes, sea surface temperature) with microfossil records (planktic and benthic foraminifera body size) to understand environmental and ecological changes during the Paleocene‐Eocene Thermal Maximum (PETM). They used these data to infer changes in oxygen levels in the tropical North Pacific and how these changes affected foraminifera body size. The study hints at the role of oxygenation in maintaining marine habitability amidst climate stress. Although deep‐sea organisms faced extinction during the PETM, those near the surface were less affected, leaving the possibility that oxygen played a role in preventing a mass extinction in the pelagic ecosystems.

These examples, as with the majority of microfossil‐based paleoecological studies, focus on the records of one taxonomic group and its relation to limited paleoclimate variables. In this area, the micropaleoecology framework can draw from approaches used in macrofossil‐based and modern ecological studies. Although such studies are often significantly limited in temporal resolution, they have leveraged cross‐taxa ecological and network metrics to provide insights into temporally restricted snapshots of communities (e.g., Sidor et al. 2013; Smith et al. 2022).

The true power of micropaleoecology lies in combining the strengths of these approaches: leveraging the high volumes of the microfossil record with metrics more commonly applied in studies of macrofossil communities and modern ecosystems with broader taxonomic coverage. Additional insights will come from systematically synthesizing across multiple fossil groups (including macrofossils, when possible), combining paleoproxies, and incorporating them into an ecosystem‐wide framework. Where past research has incorporated some aspects of the micropaleoecology framework into their pipelines, integrating all the aspects together will provide a more complete picture of paleoecological settings.

## 5 Conclusions and Future Directions

The micropaleoecology framework can facilitate investigations of ecosystem changes across multiple trophic levels and spatiotemporal scales while accounting for environmental factors. Micropaleoecology, like other emergent interdisciplinary subfields (e.g., archaeoecology, conservation paleobiology), brings together complementary data, expertises, and perspectives and can provide a common core of terminology, analyses, and thinking to grow connections between researchers. Through the integration of the detailed resolution and extensive spatiotemporal coverage of microfossil and (bio)geochemical records, we can further our understanding of variability, ecosystem resilience, and the potential ramifications of environmental and biological shifts. By incorporating ecological metrics into paleontological and abiotic records, micropaleoecology facilitates a deeper comprehension of ecosystem responses over time and space, aiding in the preparation for a future Earth facing threats from anthropogenic climate change.

## Author Contributions

**Adam Woodhouse:** writing – original draft (equal). **Anshuman Swain:** writing – original draft (equal). **Jansen A. Smith:** writing – original draft (equal). **Elizabeth C. Sibert:** writing – original draft (equal). **Adriane R. Lam:** writing – original draft (equal). **Jennifer A. Dunne:** writing – original draft (equal). **Alexandra Auderset:** writing – original draft (equal).

## Conflicts of Interest

The authors declare no conflicts of interest.

## Supporting information


Table S1.

## Data Availability

All data and code to replicate this study are available at https://github.com/anshuman21111/micropaleoecology.
